# Differential Expression of Antioxidant and Oxidant Pathways in Chronic Rhinosinusitis Without Nasal Polyps

**DOI:** 10.3390/antiox14111292

**Published:** 2025-10-28

**Authors:** Yih-Jeng Tsai, Jiunn-Min Shieh, Ming-Chieh Ma, Wen-Bin Wu

**Affiliations:** 1 Department of Otolaryngology Head and Neck Surgery, Shin Kong Wu Ho-Su Memorial Hospital, Taipei 111045, Taiwan; 083100@mail.fju.edu.tw; 2 School of Medicine, Fu Jen Catholic University, New Taipei City 242062, Taiwan; med0041@mail.fju.edu.tw; 3 Department of Internal Medicine, Chi Mei Medical Center, Tainan 710832, Taiwan; shieh225116@gmail.com; 4 Graduate Institute of Biomedical and Pharmaceutical Science, Fu Jen Catholic University, New Taipei City 242062, Taiwan

**Keywords:** CRSsNP, DEGs, OxS, RNS, ROS

## Abstract

Chronic rhinosinusitis without nasal polyps (CRSsNP) is a chronic inflammatory disease that lacks a clear pathogenesis/pathophysiology. While large studies focused on elucidating the pathophysiology of CRS with NPs (CRSwNP), this study aimed to use a systemic evaluation approach to identify the redox gene expression profile, its association with oxidative damage in CRSsNP, and the differences between CRSsNP and -wNP. The expression of 84 redox genes was analyzed using real-time PCR array in control and CRSsNP nasal mucosae. Changes in the mRNA and protein levels of these redox differentially expressed genes (DEGs) were verified using a customized real-time PCR array, RT-PCR, and Western blotting in an additional 18 patients. 4-Hydroxynonenal (lipid peroxidation) and 3-nitrotyrosine (protein nitrosylation) expression, representing oxidative stress (OxS) and nitrosative stress (NsS) status, were examined using immunohistochemistry. We found 27 DEGs (24 upregulated and 3 downregulated) in CRSsNP. AKR1C2, GCLM, GPX2, NOS2, and NQO1 were upregulated and LPO was downregulated more than 4-fold. These changes led to a substantial increase in OxS in CRSsNP nasal mucosa. In a comparison of the currently identified 27 DEGs with the 23 previously reported CRSwNP genes, there were 16 unique redox DEGs expressed between CRSsNP and -wNP. A String protein interaction network analysis revealed that CRSsNP possessed “an adaptive antioxidant defense signature”, while CRSwNP showed “a pro-inflammatory and -oxidant pathway”. Collectively, we systemically performed transcriptomic analysis to profile OxS-related genes in CRSsNP and highlighted the unique redox gene sets and pathway differences between CRSsNP and -wNP.

## 1. Introduction

Oxidative stress (OxS) is a phenomenon caused by an imbalance between the production and accumulation of reactive oxygen/nitrogen species (ROS/RNS) in cells and tissues and the ability of a biological system to detoxify these reactive products [1,2]. Superoxide radicals (O_2_^•−^), hydrogen peroxide (H_2_O_2_), hydroxyl radicals (^•^OH), and singlet oxygen (^1^O_2_) are commonly defined as ROS [3], while nitric oxide (NO^•^), peroxynitrite (ONOO^−^), S-nitrosothiols (RSNOs), and others are RNS [4]. The accumulation of ROS/RNS-induced damage, including damage to cellular molecules such as DNA, proteins, and lipids, is responsible for the development of diseases, especially chronic inflammatory diseases [1,5]. In contrast, the antioxidant defense system of cells, mainly consisting of enzymes such as superoxide dismutase (SOD), catalase (CAT), and glutathione peroxidase (GPx), protects them from ROS/RNS-induced damage through antioxidant effects [6].

Chronic rhinosinusitis (CRS) can be subdivided into two major categories, CRS with nasal polyps (CRSwNP) and without nasal polyps (CRSsNP), based on the phenotypes of whether nasal polyps (NPs) are present or absent [7,8]. CRSsNP is characterized by mainly Th1-driven inflammation with high levels of IFN-γ and active TGFβ1 signaling with fibrosis, basement membrane thickening, goblet cell hyperplasia, subepithelial edema, and mononuclear cell infiltration [9]. The mechanism of action of remodeling in CRSsNP is still unclear. Therefore, a better understanding of CRSsNP pathogenesis/pathophysiology is needed to advance the current diagnosis and treatment of these patients.

While extensive studies have focused on OxS in CRSwNP [10,11,12,13,14], less attention has been paid to CRSsNP. A significant difference has been reported in the number of microplastics, which has been shown to cause oxidative damage and neurotoxicity between the CRSsNP and the control group [15]. The expression in the apical portion of the nasal epithelium of dual oxidases, which are responsible for H_2_O_2_ generation in the airway epithelium, is increased in CRSwP and CRSsP [16]. However, when comparing the control patients to their secondhand smoke (SHS)-exposed counterparts, SHS exposure was associated with statistically significantly higher levels of ROS-positive cells. The SHS exposure does not affect ROS levels in CRSsNP and CRSwNP patients [17]. No correlation was observed between any glutathione S-transferases polymorphism and CRSwNP/sNP [18].

In our previous study, the expression of OxS-related genes in nasal mucosae and NP tissues was analyzed via PCR microarray analysis. We found that 19 genes were significantly upregulated, and 4 were significantly downregulated. Among them, inducible nitric oxide synthase (iNOS) and heme oxygenase 1 (HO-1) were notably upregulated, whereas lactoperoxidase (LPO), myeloperoxidase (MPO), and superoxide dismutase 3 (SOD3) were highly downregulated [12]. In this study, we aimed to determine the oxidant/antioxidant status and gene expression profile in CRSsNP through the systemic transcriptomic analysis approach. Among the 84 OxS genes tested, 24 were overexpressed, while 3 were underexpressed. The results were confirmed by a customized real-time PCR array and RT-PCR analysis, as well as Western blotting. An apparent increase in OxS, including lipid peroxidation and protein tyrosine nitrosylation, was found in CRSsNP. Interestingly, further analysis identified 16 redox differentially expressed genes (DEGs) expressed between CRSsNP and -wNP and revealed that CRSsNP possessed “an adaptive antioxidant defense signature”, whereas CRSwNP displayed “a pro-inflammatory and oxidant pathway”.

## 2. Materials and Methods

### 2.1. Materials

The Ab raised against NOS2 (iNOS) (catalog no: 610328) was obtained from BD Biosciences (Becton Drive-Franklin Lakes, NJ, USA). The heme oxygenase-1 (HMOX1; HO-1) (catalog no: ab13243), superoxide dismutase 3 (SOD3) Ab (catalog no: ab21974), and lactoperoxidase (LPO) Ab (catalog no: ab231026) were purchased from Abcam (Cambridge, MA, USA). The Ab for β-actin (catalog no: GTX110564) was purchased from GeneTex, Inc. (Hsinchu, Taiwan).

### 2.2. Patient Recruitment and Sample Collection

This study was approved by the Ethics Committee of the Shin Kong Wu Ho-Su Memorial Hospital, Taipei, Taiwan (Permission No: 20161210R), and informed consent was obtained from the patients. The nasal mucosa tissues from 24 patients with CRSsNP were collected for this study. CRSsNP was diagnosed based on patient history, local findings from anterior rhinoscopy, nasal endoscopy, and sinus computed tomography. No patient had a history of allergy, asthma, or aspirin sensitivity and none had been treated with either oral or topical antiallergic agents or steroids for at least 2 months. The ethmoidal mucosa and the mucosa around the osteomeatal complex in the CRSsNP group were retrieved during functional endoscopic sinus surgery. In the control group, patients with blockages in their lacrimal drainage systems were free of other nasal diseases, and the control agger nasi sinus cell mucosae were prepared during dacryocystorhinostomy procedures [12]. The patients’ demographic data and the tissue samples used for this study are summarized in Table S1. Briefly, nasal tissue samples were taken from 6 control and 6 CRSsNP patients for the 84-gene real-time PCR microarray analysis. Additionally, 18 independent control and CRSsNP patients’ samples were used for the customized real-time PCR microarrays. The remaining tissue samples from these 24 patients were randomly used for RT-PCR, Western blot, and immunohistochemistry analysis.

### 2.3. Real-Time PCR Microarrays

We utilized real-time PCR microarray analysis to map the complete gene expression related to OxS. Total RNAs of human and CRSsNP control nasal mucosae were isolated by using an Rneasy Mini kit (Qiagen, Valencia, CA, USA). The cDNA was transcribed using an RT^2^ Reaction Ready First Strand Synthesis Kit (Qiagen) and was analyzed using the human OxS PCR array (Qiagen). The real-time PCR microarrays used an RT^2^ SYBR Green Fluor qPCR Mastermix (Qiagen) on a 7300 Real-Time PCR System (Applied Biosystems, Foster City, CA, USA). Data were normalized by using housekeeping genes (β-actin, glyceraldehyde-3-phosphate dehydrogenase (GAPDH), β2-microglobulin (B2M), ribosomal protein, large, p0 (RPLP0)) and analyzed by comparing the 2^−ΔΔCt^ of the normalized sample. For confirmation/validation of the (non-)altered genes by customized real-time PCR microarrays, we conducted a customized human OxS PCR array from Qiagen. A total of 18 additional and independent human control and CRSsNP nasal tissue samples were used in this confirmation. The specific primers were designed to detect the expression of the non-changed and some significantly altered genes alongside two housekeeping genes, β-actin and RPLP0. The methodology was consistent with the above real-time PCR microarray protocol.

### 2.4. RT-PCR Analysis of mRNA Expression Levels of DEGs

Changes in the mRNA expression levels of some of the significantly differentially expressed genes observed in the microarrays were examined by using RT-PCR for the individual transcripts. The tissues were processed as homogenates. The primers used are listed in Table 1. Total RNA extraction, 1^st^-strand cDNA synthesis, and PCR analysis were performed as previously described [19], except the annealing temperature for the PCR was set to 51–61 °C, depending on the sequences of the primers used. 

### 2.5. Tissue Lysate Preparation and Western Blot Analysis

Nasal tissue lysates were prepared as previously described [20]. Total proteins were analyzed on SDS–polyacrylamide gels, electroblotted onto PVDF membranes (EMD Millipore Corporation, Billerica, MA, USA), and then probed using a primary Ab. The immunoblots were developed using Immobilon Western Chemiluminescent HRP Substrate (EMD Millipore). The membranes were stripped with a stripping buffer, washed, and reprobed with the Abs to examine the level of β-actin and then developed.

### 2.6. Immunohistochemistry

The modifications of 4-hydroxynonenal (4-HNE) and 3-nitrotyrosine in both control and CRSsNP nasal mucosae were assessed using immunohistochemistry (IHC), following a previously described method with slight modifications [21]. In brief, tissue sections were deparaffinized and hydrated in graded ethanol. After washing, the sections were immersed and heated in a water bath for 20 min. The slides were then incubated overnight at 4 °C with primary antibodies specific to 4-HNE or 3-nitrotyrosine (Abcam), following a blocking step with a buffer containing 10% FBS (Thermo Fisher Scientific, Waltham, NY, USA). After washing with TBS, the slides were treated with Super Enhancer and Poly-HRP and developed using one-step 3-amino-9-ethylcarbazole with the Super Sensitive Polymer-HRP IHC Detection System (Biogenex Laboratories, Inc., Fremont, CA, USA) for 5–30 min. Finally, the sections were counterstained with hematoxylin for 20–40 s, washed with tap water, and mounted using 100% glycerol. The quantitation of the staining results was performed using the Invitrogen Celleste 5.0 Image Analysis Software (Thermo Fisher Scientific, Waltham, MA, USA). Three random regions of interest (ROIs) per nasal mucosa were analyzed per patient. Mean optical density (OD) was computed from the positively stained area relative to the total ROI area.

### 2.7. Data Analysis

The PCR microarray data underwent a normality test per sample by using the Shapiro–Wilk test, which indicated they followed normal distribution. Protein–protein association networks and functional enrichment analyses of the DEGs were performed using STRING database (version 12.0; https://string-db.org/) [22]. All data are expressed as the mean ± SEM unless otherwise indicated. Differences between groups were compared with an unpaired and parametric Student’s *t*-test. Differences were considered to be statistically significant at *p* < 0.05.

## 3. Results

### 3.1. Twenty-Seven OxS-Related Genes Are Significantly Altered in CRSsNP Nasal Mucosa Tissues

To explore the changes in OxS-related genes in CRSsNP, a quantitative real-time PCR microarray analysis was performed to analyze 84 genes in the nasal mucosa tissues of control and CRSsNP tissues. Gene expression changes greater than two-fold with a *p*-value of <0.05 were considered to be significantly differentially expressed in CRSsNP patients. A total of 27 genes were significantly differentially expressed and are designated differentially expressed genes (DEGs), as shown in Table 2. Among the DEGs, 24 were overexpressed (green text/number) and 3 were underexpressed (red text/number). The overexpressed genes were ALB, AKR1C2, BAG2, DUOX1, EPX, GCLC, GCLM, GPX2, GSTP1, HSP90AA1, HMOX1, NQO1, NCF2, NOS2, PRDX1, PRDX3, PRDX5, SLC7A11, SOD1, TXN, TXNRD1, TPO, TTN, and UCP2, and the underexpressed genes were GPX3, LPO, and SOD3.

**Figure 1:**
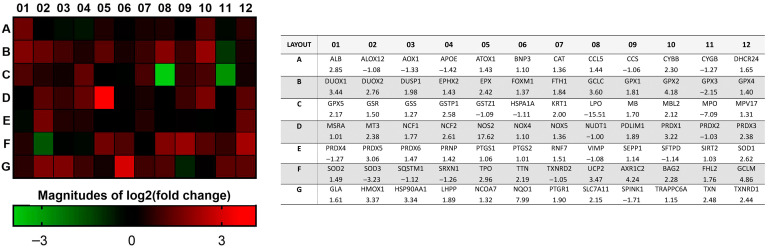
The fold change and regulation in the OxS gene expression between the control and CRSsNP nasal mucosa tissues. The heat map provides log2 (fold change), whereas the table provides fold regulation data. Note that AKR1C2, GCLM, GPX2, NOS2, NQO1, and LPO were DEGs that changed by more than 4 times.

**Figure 2:**
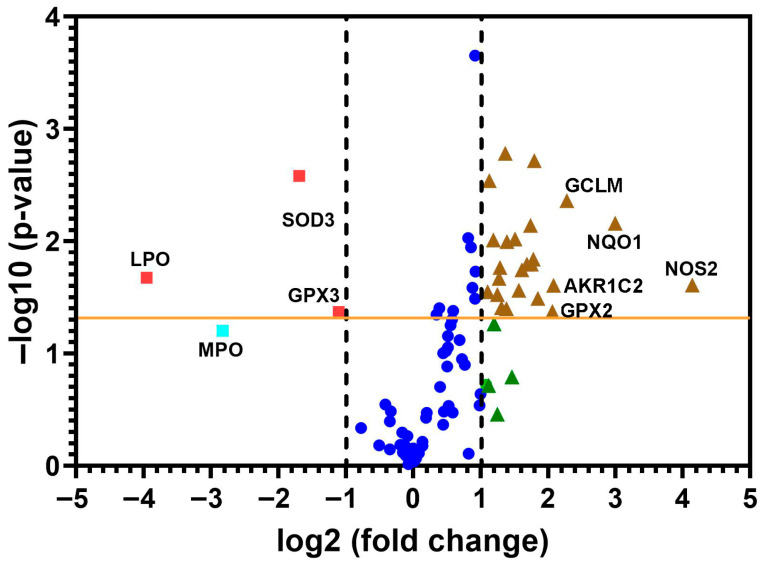
The volcano plot of the changes in redox gene expressions. The 84 genes were examined from the control and CRSsNP nasal mucosa linked to OxS. The y-axis represents statistical significance, and the x-axis shows fold changes. Two vertical dashed black lines mark the threshold for two-fold changes in gene expression, and the orange line indicates a *p*-value of 0.05. Genes that showed significant changes are positioned beyond the dashed lines and above the orange line (denoted by brown triangles and red squares). The uDEGs that changed by more than 4 times and all dDEGs were labeled with their respective gene name. Deep blue circles represent genes less than two-fold changes, whereas green triangles and a light blue square are genes more than two-fold changes without statistical significance.

A heat map of the 84 genes provided a whole view of the fold changes in their expression between the control and CRSsNP groups (Figure 1). The two markedly upregulated DEGs (uDEGs) were inducible nitric oxide synthase (iNOS; NOS2) and NAD(P)H dehydrogenase, quinone 1 (NQO1), which were upregulated by more than 17.62 and 7.99 times, respectively. Conversely, the most downregulated DEG (dDEG) was LPO, which was underexpressed by more than 15.51-fold. The uDEGs with greater than 4-fold changes were AKR1C2, GCLM, GPX2, NOS2, and NQO1, whereas the only dDEG with more than 4-fold change was LPO. All of the significant DEGs in CRSsNP that had a greater than two-fold change (black and dashed lines) with a *p*-value < 0.05 (above the orange line) are shown in a volcano plot (Figure 2).

### 3.2. Confirmation of the DEGs by RT-PCR, Western Blot, and Customized Real-Time PCR Array Analyses

**Figure 3:**
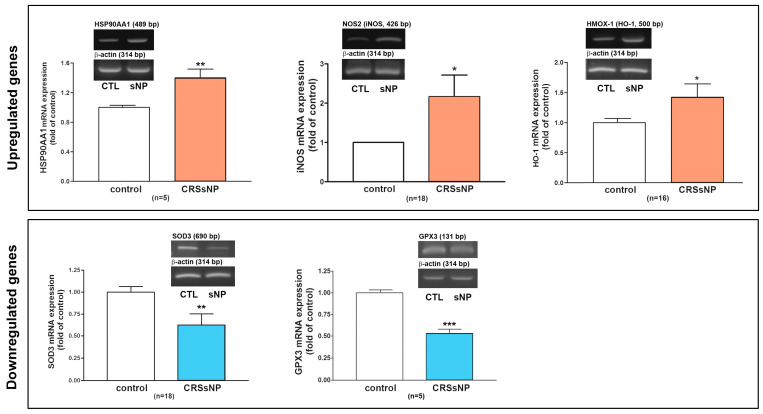
RT-PCR analysis to verify the mRNA expression of some DEGs. The total RNA was extracted from human control and CRSsNP nasal mucosa tissues and the mRNA expression levels of the indicated genes were determined by RT-PCR analysis. Data are mean ± SEM. CTL: control; sNP: CRSsNP. * *p* < 0.05, ** *p* < 0.01, and *** *p* < 0.001 versus control.

Next, an RT-PCR analysis of the selected individual transcripts of the genes was performed. The data confirmed the significant upregulation of mRNA expression of HSP90AA1 (1.5-fold increase), iNOS (2.1-fold increase), and HMOX1 (also called HO-1) (1.4-fold increase) and the significant downregulation of mRNA expression of SOD3 (reduced to 0.67-fold of control) and GPX3 (reduced to 0.5-fold of control) in the CRSsNP nasal mucosa tissues (Figure 3).

**Figure 4:**
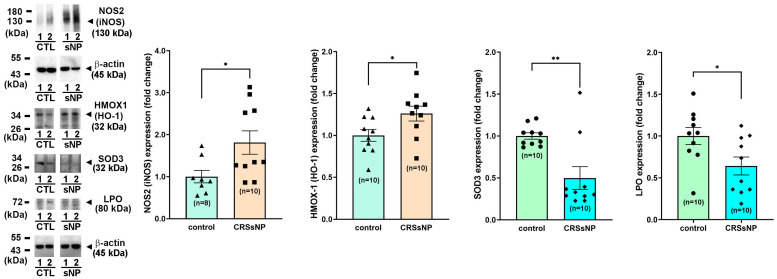
The protein expression levels of the randomly selected DEGs. The protein levels of the altered genes in the control and CRSsNP nasal mucosae were analyzed by Western blotting followed by densitometric analysis. The representative and quantitative results are shown (*n* = 8–10 for control and *n* = 10 for CRSsNP). Data are mean ± SEM. * p < 0.05 and ** p < 0.01 versus control.

To further investigate whether the protein DEG products are correspondingly changed in the nasal mucosa tissues of CRSsNP, the control and CRSsNP nasal mucosae were analyzed via Western blot (WB) analysis. Figure 4 shows two representative gel images for NOS2 (130 kDa in a non-reduced form), HMOX1 (32 kDa), SOD3 (32 kDa), LPO (80 kDa), and β-actin (45 kDa) expression in the nasal mucosae of controls and CRSsNP patients via WB analysis (left panels). The quantitative densitometric analysis of all collected samples (*n* = 8–10) indicated significantly increased levels of both NOS2 and HMOX-1 in the CRSsNP nasal samples, with approximately 1.8- and 1.3-fold increases, respectively. A reduced expression of SOD3 and LPO protein was found in CRSsNP, with about 0.5- and 0.6-fold of control (right panels). This indicates that the DEGs’ corresponding proteins were simultaneously up- and downregulated.

Next, the two non-significantly changed genes (DUSP1 and PRDX4) and four DEGs (LPO, SOD3, NOS2, and HMOX1) were further selected to confirm and validate the DEGs in 18 additional patients’ nasal tissue samples using the customized real-time PCR array analysis (Table 3), confirming that the non-significantly changed genes remained unchanged, and the DEGs remained significantly changed.

### 3.3. The DEGs Lead to the Presence of Oxidative/Nitrosative Damage in CRSsNP Nasal Mucosa

We have identified 24 and 3 genes to be up- and downregulated, respectively, in CRSsNP. An in-depth literature search was performed to analyze the possible effects of ROS (Table 4a) and RNS (Table 4b) on antioxidant defense and prooxidant activity in tissues in relation to these DEGs. Briefly, as shown in Table 4a, the overexpressed PRDX1/3/5, GPX2, SOD1, TXN, and TXNRD increase ROS detoxification [23], and the GCLC, GCLM, and SLC7A11 enhance glutathione (GSH) synthesis [24]. In contrast, the downregulated GPX3 and SOD3 decrease extracellular ROS clearance and can increase extracellular OxS risk [25,26], and the upregulated DUOX1, NOS2, and NCF2 can lead to ROS/RNS generation, including H_2_O_2_, NO, and superoxide [27]. The increased EPX causes oxidative burst activity, and decreased LPO expression lowers H_2_O_2_ conversion (H_2_O_2_↑) and antimicrobial defense [28]. Table 4b shows that NOS2 can be a direct NO donor and enhances RNS production [29]. GCLC, GCLM, SLC7A11, TXN, and TXNRD1 serve as an S-nitrosoglutathione (GSNO) pool [24,30], and TXN and TXNRD1 mediate protein trans-nitrosylation and denitrosylation [31]. Moreover, DUOX1, NCF2, EPX, and CYBB mediate ROS-NO crosstalk [32]. These could contribute to RNS production, nitrosyl group formation, and protein nitrosylation.

**Figure 5:**
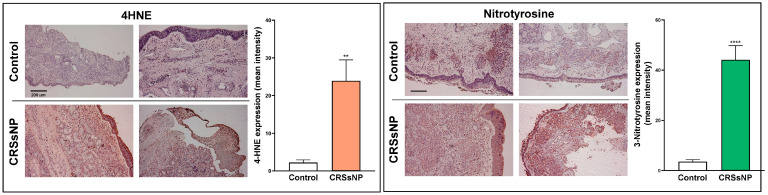
The increase in OxS and NsS in CRSsNP nasal mucosae. The control and CRSsNP (SNP) nasal mucosae were analyzed by IHC to determine the status of OxS and NsS by using anti-4-HNE Ab, anti-3-nitrotyrosine Ab, and their corresponding nonimmune IgG (NIgG). The images were captured under a phase-contrast microscope. A (deep) red color indicates positive staining of 4-HNE or 3-nitrotyrosine. Scale bar: 200 μm. The quantitation of the staining results was performed by measuring the mean intensity of three positive staining areas of each tissue sample using the Invitrogen Celleste 5.0 Image Analysis Software (*n* = 9). ** *p* < 0.01 and **** *p* < 0.0001 versus control.

The analysis in Table 4 only reflects the possible imbalance of antioxidant defense and ROS/RNS-mediated OxS damage due to DEGs, but their association with the overall OxS status in CRSsNP nasal tissue samples still needs to be explored. It has been reported that the various degradation products of lipid peroxidation include α- and β-unsaturated hydroxyalkenal, as well as 4-hydroxy-2,3-trans-nonenal (4-HNE) [33], whereas 3-nitrotyrosine results from the nitration of both protein-bound and free tyrosine residues by reactive peroxynitrite molecules [34]. Therefore, 4-HNE and 3-nitrotyrosine modifications were assayed via IHC using the respective specific antibodies. As shown in Figure 5, while only a little 4-HNE staining was found in the control nasal mucosae, a lot of positive staining for 4-HNE (deep red color) was observed in the CRSsNP nasal mucosa tissues. Positive staining was found in the epithelium, subepithelial stromal cells, glands, and some infiltrated leukocytes. Meanwhile, the 3-nitrotyrosine modifications were also enhanced in the CRSsNP nasal mucosae, which were located in the infiltrated leukocytes underneath the epithelium infiltrated leukocytes but were expressed in the epithelium, glands, and some infiltrated leukocytes. Taken together, our results reveal a significant increase in oxidative and nitrosative stress in CRSsNP, including lipid peroxidation and protein tyrosine nitrosylation.

### 3.4. The Analysis by STRING Reveals a Novel Adaptive Antioxidant Defense Imbalance Signature in CRSsNP but a Pro-Inflammatory/Oxidant Pathway in CRSwNP

**Figure 6:**
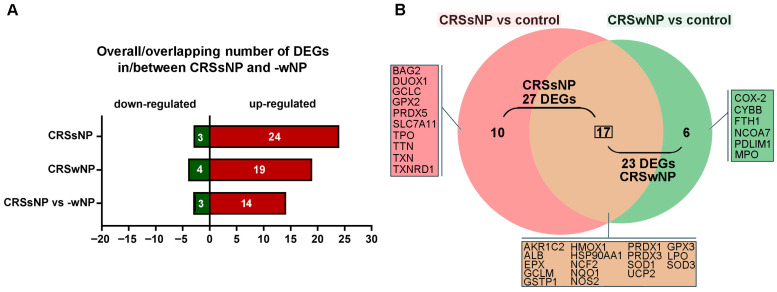
Analysis of OxS-related DEGs in CRSsNP and -wNP. (**A**) Overall/overlapping DEGs (Up- and downregulated genes) in/between nasal tissue samples of CRSsNP versus control and CRSwNP versus control. (**B**) The Venn diagram shows the number and genes of (un)intersective DEGs between CRSsNP versus control and CRSwNP versus control.

The gene expression profile in CRSsNP was compared with that found in CRSwNP [12]. A shown in Figure 6A, 24 uDEGs and 3 dDEGs were found in CRSsNP, whereas 19 and 4 were identified in CRSwNP [12]. In Figure 6B, the Venn diagram shows that there were 17 DEGs completely identical/overlapping in CRSsNP and -wNP. However, 16 DEGs were quite different between CRSsNP and -wNP, in which 10 DEGs were unique in CRSsNP and only 6 DEGs were particular to CRSwNP (panel B). To further pinpoint the DEG association with OxS in CRSsNP and -wNP, the total 27 and 23 DEGs of CRSsNP and -wNP were subjected to analysis by the STRING protein interaction network and gene ontology (GO) enrichment analysis. In Figure S1, a highly dense antioxidant hub was found in CRSsNP. Moreover, the presence of GCLC, GCLM, GSTP1, TXNRD1, and GPX2 in CRSsNP highly suggested strong GSH- and thiol-based antioxidant detoxification potential. On the contrary, in wNP, the CYBB (NOX2), MPO, and PTGS2 were tightly connected to iron metabolism (FTH1, HMOX1), favoring an inflammatory oxidative burst (panel A). The GO enrichment analysis indicated that CRSsNP and -wNP shared two identities in the top three biological processes and four identities in the top five molecular functions. It was noted that the “response to toxic substance” and “thioredoxin peroxidase activity” were unique in CRSsNP in GO biological process and molecular function (panel B, box and highlighted region).

**Figure 7:**
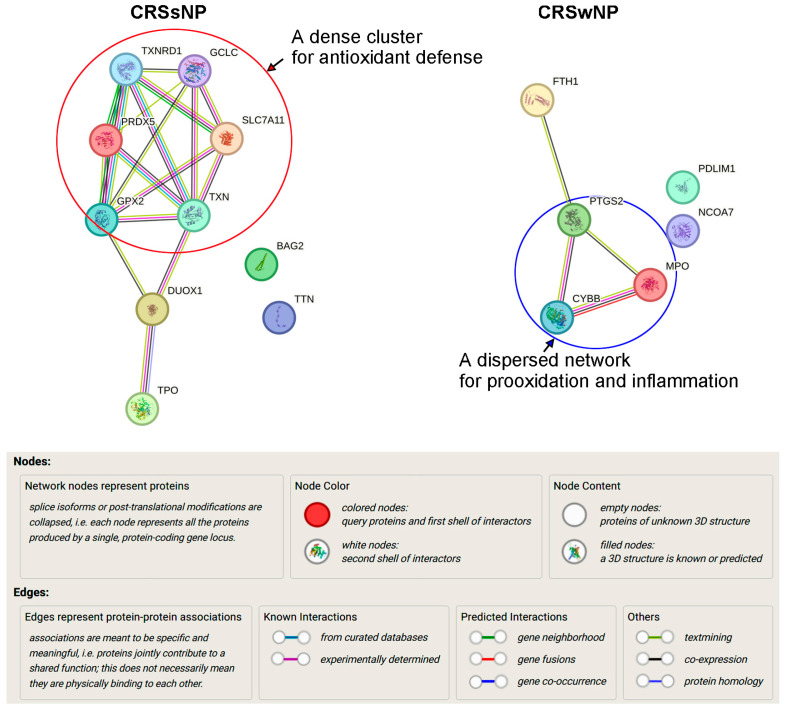
Analysis of protein–protein interaction. The 10 and 6 distinct DEGs of CRSsNP and -wNP obtained from the Venn diagram were subjected to analysis by the STRING Database (Version 12.0) for determining the protein–protein interactions. The explanation of the nodes, lines, and edges in the interaction network is provided. The circles highlighting the proteins located in the core cluster/network of the interactions suggest an antidefense pathway in CRSsNP but a prooxidant and inflammatory pathway in CRSwNP.

Surprisingly, if the overlapping 17 DEGs were excluded and only the unique 10 and 6 DEGs in CRSsNP and -wNP were, respectively, subjected to analysis by the STRING, further differences were revealed. In Figure 7, in CRSsNP, the cluster genes, like TXNRD1, PRDX5, TXN, GPX2, GCLC, SLC7A11, formed a highly interconnected and tight core network, indicating strong functional interdependence, especially within antioxidant defense pathways including the thioredoxin system (TXN, TXNRD1), peroxiredoxin system (PRDX5), and GSH system (GCLC, GPX2, SLC7A11) (highlighted by a circle). The peripheral yet connected DUOX1 and TPO reflect a separate role in extracellular ROS production and possibly in thyroid hormone biosynthesis, which likely bridged intracellular ROS detoxification and extracellular ROS generation roles. The isolated nodes (BAG2, TTN) suggest different or secondary roles in CRSsNP and may be non-redox-related. Therefore, the network suggests “a dominant, adaptive antioxidant defense signature” with additional roles in extracellular redox signaling. Conversely, in CRSwNP, the core triangle cluster of PTGS2, MPO, and CYBB (NOX2) with FTH1 at the top indicates centrality in ROS/RNS generation (CYBB via NADPH oxidase and PTGS2) and neutrophil- and prooxidant-mediated microbial killing (MPO) with iron metabolism (FTH1) bridging the gap, suggesting “a prooxidant-inflammatory axis” (highlighted by a circle). Both PDLIM1 and NCOA7 were unconnected and might play non-core roles, perhaps linked to transcriptional regulation or structural modulation under CRSwNP.

## 4. Discussion

Low levels of ROS/RNS production are required to maintain physiological functions such as signal transduction and host defense, but a higher level of ROS/RNS due to an imbalance between oxidant and antioxidant defense systems can produce OxS, damaging cellular macromolecules [35,36]. In this study, we demonstrated that out of the 84 genes examined, 24 and 3 OxS-related genes were significantly up- and downregulated in CRSsNP, respectively (Table 2). The results were confirmed and validated by the customized PCR array, RT-PCR, and Western blotting (Table 3 and Figure 3 and Figure 4). Further analysis suggested participation of these DEGs in prooxidant (ROS/RNS production) and antioxidant defense (Table 4), and OxS was higher in CRSsNP nasal mucosae, indicating that the DEGs substantially led to an increase in OxS and NsS in CRSsNP (Figure 5). Notably, in comparison with the findings of our previous study [12], we not only identified the unique genes for CRSsNP and -wNP but also demonstrated that CRSsNP possesses “a dominant, adaptive antioxidant defense signature”, while CRSwNP prefers “a pro-inflammatory and oxidant pathway” (Figure 6, Figure 7 and Figure S1).

Regarding the distinct signature/pathway in CRSsNP and -wNP, it was found that a total of 16 unique DEGs were distinct between them (Figure 6). In sNP, these DEGs, such as GPX2, PRDX5, TXN, and TXNRD1, are all central to reducing ROS production [23,26,37,38], and GCLC and SLC7A11 are crucial in GSH biosynthesis and cystine uptake, which enhances cellular redox buffering capacity [24,39], suggesting strong intracellular antioxidant reinforcement, especially via GSH and thioredoxin systems. On the other hand, CYBB (as a NOX2 subunit), PTGS2 (COX-2), FTH1, and PRDX3 formed a linear connection in wNP, highly suggesting prooxidant pathways and inflammation as COX-2 produces prostaglandins (inflammatory ROS) [40], CYBB generates superoxide in phagocytes [41], FTH1 (Ferritin Heavy Chain) regulates iron storage and prevents Fenton reaction-mediated oxidative damage [42,43], and NCOA7 has oxidant detoxification roles in lysosomes [44,45]. Therefore, it can be concluded that CRSsNP has a signature of adaptive antioxidant responses, whereas CRSwNP has highlighted inflammatory ROS production (prooxidant) pathways and perhaps less reliance on antioxidant reinforcement. Nevertheless, one may question such a signature, as the presence of OxS and NsS in CRSsNP seems contradictory. This can be explained by the inconsistently translated protein amounts of these DEGs and a compensatory (secondary) antioxidant response to the ongoing OxS, which is known as “antioxidant response element (ARE)-driven compensation”, especially by Nrf2 [46]. Moreover, the signature may involve a tissue protective mechanism to OxS and NsS.

Among the overlapping DEGs between CRSsNP and -wNP, the upregulated NOS2, NQO1, AKR1C2, GPX2, and GCLM and the downregulated LPO were notable and showed more than 4-fold changes. NOS2 is known as an inducible NO synthase (iNOS) and is well known to produce NO [47]. There are many studies indicating that NOS2 is more highly expressed in NPs than in normal cases [48,49,50,51,52]. However, few studies have reported overexpression of NOS2 in CRSsNP. Recently, it was shown that CRSwNP patients exhibited decreased nasal NO despite elevated NOS2 mRNA expression, implying that lowered nasal NO production in CRSwNP may not be related to NOS expression [51]. This may raise a question about whether the increase in NOS2 expression correlates with the nasal NO production in CRSsNP. This is highly speculative, since NOS2 is a major donor for NO and RNS and protein nitrosylation has been found to be higher in CRSsNP (Figure 5). NQO1, belonging to the NAD(P)H dehydrogenase (quinone) family, is a FAD-binding protein family that prevents the one-electron reduction in quinones that results in the production of radical species [53,54]. It has been shown that NQO1 knockdown enhances ROS production and diminishes cell proliferation, but its overexpression increases proliferation in glioblastoma cells [55]. Moreover, GPX2 belongs to the glutathione peroxidase family [26]. These gene products are responsible for removing ROS and other harmful substances and facilitating anti-OxS. As to GCLM and AKR1C2, they are the first-rate limiting enzymes of GSH synthesis and are involved in eliminating ROS and regulating tumor invasion, migration, and other malignant phenotypes [56]. Among these upregulated DEGs, HMOX-1 and GPX2 were located at the core of network interaction, whereas AKR1C2, GCLM, NQO1, and NOS2 were at the periphery (Figure S1). The reasons why LPO was downregulated in CRSsNP and -wNP remain to be explored. LPO is synthesized and secreted from epithelial cells [28,57]. It is unlikely that the downregulation resulted from shedding/damage of the epithelium. Our recent unpublished data found that a bacterial cell wall component downregulates basal constitutive LPO production in nasal epithelial cells, suggesting that its expression can be tightly regulated in nasal epithelial cells in a CRS microbial infection microenvironment.

Our analysis of the literature indicated the putative pro- and antioxidant activities of 27 dysregulated DEGs (Table 4), and OxS and NsS levels were demonstrated to be higher in the CRSsNP nasal mucosae (Figure 5). The reaction between O_2_^•−^ and nitric oxide (NO^•^) may produce ONOO^−^, and their decomposition then causes some highly oxidizing intermediates, including NO_2_^•^, OH^•^, CO_3_^•−^, and NO_3_^−^ [58,59]. The nitric oxide and peroxynitrite molecules contribute to the nitrosylation of protein tyrosine [34]. The upregulation of DUOX1, NCF2, and NOS2 (iNOS) may contribute to ROS, O_2_^•−^, and NO production. For example, NCF2 is part of the NADPH oxidase complex (NOX2) and can produce superoxide, which reacts with NO to form peroxynitrite [4]. BAG2 has been shown to protect neurons against 1-methyl-4-phenylpyridinium-induced OxS in an in vitro cell model of Parkinson’s disease [60]. In addition, numerous initiators of lipid peroxidation, such as 4-HNE in biological systems, are often hydroxyl radical (OH^•^), ozone (O_3_), nitrogen oxide (NO), nitrogen dioxide (NO_2_), and sulfur dioxide (SO_2_) [61], which may result from the downregulation of GPX3, LPO, and SOD3. The increase in ROS production may also lead to the upregulation of several antioxidants to clear ROS, such as TXN and TXNRD1 (reduction in disulfides (S-S) within oxidized cellular proteins), PRDX1, 3, and -5 (detoxifying H_2_O_2_), HMOX-1 (degradation of cellular heme against OxS), GCLM, AKR1C2, SLC7A11, NQO1, and GSTP1 for GSH synthesis and function.

It may be a limitation that this study used a small number of six control and CRSsNP samples for transcriptomic profiling (PCR microarray analysis), but the effect sample sizes were calculated to be 10, 21, and 37 based on a formal power analysis (see the calculations in Supplementary data). In this regard, six of the (non)DEGs were further confirmed and validated by the customized PCR microarray with an “independent” 18 patients (cohort). We showed the same (un)changed trend in these genes as our initial profiling and with smaller *p*-values in an appropriate effect sample size (>*n* = 14 by the formal power analysis with σ = 0.75). Due to limited tissue specimens, it should be mentioned that the sample size in initial profiling did not reach the calculated effect size. The small size may limit power [only detects very large but may miss moderate effects (false negatives)] and reduce robustness. In addition, the small set of significant hits may be overestimated. The authors would also like to address the limitation of using different sinus subsites for control and CRSsNP samples (which have different epithelial cell compositions and basal gene expression), while most studies adopt nasal mucosae from nasal septum deviation surgery and agger nasi mucosa removed during dacryocystorhinotomy as a control.

For a quick overview of our findings, a side-by-side table is provided to summarize the overlapping and unique DEGs, their biological functions, and possible clinical implications (Table S2). Our STRING analysis suggested CRSwNP with a prooxidant–inflammatory axis, which is more closely associated with type 2 inflammatory pathways and explained the favorable response of these patients to biologic therapies and surgery [62,63]. However, an important clinical nuance is that a subset of CRSsNP patients may also exhibit type 2-high endotypes despite the absence of visible NPs [64]. This biological heterogeneity may help explain the remarkable response to biologic therapies observed in some CRSsNP patients. Therefore, the findings of this study highlight the need for endotype-driven treatment approaches rather than relying solely on the phenotypic classification of CRS.

## 5. Conclusions

In this study, we used a systems biology approach to profile OxS-related genes and identified 27 DEGs and the status of oxidative stress in CRSsNP nasal mucosa tissues. The DEGs were verified and confirmed by a customized PCR microarray, RT-PCR, and Western blotting. More importantly, we compared the DEGs in CRSsNP with previously reported CRSwNP DEGs and found up to 16 genes distinct between them. We showed here that CRSsNP possesses “an adaptive antioxidant defense signature”, while CRSwNP tends to exhibit “a pro-inflammatory/oxidant pathway”, highlighting the unique redox gene sets and pathway differences between CRSsNP and -wNP.

## Figures and Tables

**Table 1:** Primers for reverse transcription polymerase chain reaction.

Gene	Forward Primer (5′ ⟶ 3′)	Reverse Primer (5′ ⟶ 3′)	Product Size
*HSP90AA1*	GCCGGATGCCTAAGTAGACC	CCATGTCAACCCTTGGAGCA	489 bp
*NOS2*	GCAGAATGTGACCATCATGG	ACAACCTTGGGGTTGAAGGC	426 bp
*HMOX1*	GAGACGGCTTCAAGCTGGTGATG	GTTGAGCAGGAACGCAGTCTTGG	500 bp
*SOD3*	ATGCTGGCGCTACTGTGTT	GCTTCTTGCGCTCTGAGT	690 bp
*GPX3*	GCCGGGGACAAGAGAAGT	GAGGACGTATTTGCCAGCAT	131 bp
*β-ACTIN*	ATCATGTTTGAGACCTTCAA	CATCTCTTGCTCGAAGTCCA	314 bp

**Table 2:** PCR microarray analysis of 84 OxS genes involved in CRSsNP.

Gene Ref No.	Gene Name/Abbreviation	Fold Regulation	*p*-Value (*p* < 0.05)
NM_014762	24-Dehydrocholesterol reductase (DHCR24)	1.65	0.112073
NM_000477	Albumin (ALB)	2.85	0.00957
NM_001159	Aldehyde oxidase 1 (AOX1)	−1.33	0.285081
NM_001354	Aldo-keto reductase family 1, member C2 (dihydrodiol dehydrogenase 2; bile acid binding protein; 3-alpha hydroxysteroid dehydrogenase, type III) (AKR1C2)	4.24	0.024788
NM_000041	Apolipoprotein E (APOE)	−1.42	0.660256
NM_000697	Arachidonate 12-lipoxygenase (ALOX12)	−1.08	0.814280
NM_004045	ATX1 antioxidant protein 1 homolog (yeast) (ATOX1)	1.43	0.088614
NM_004052	BCL2/adenovirus E1B 19kDa interacting protein 3 (BNIP3)	1.10	0.612231
NM_004282	BCL2-associated athanogene 2 (BAG2)	2.28	0.009770
NM_001752	Catalase (CAT)	1.36	0.099366
NM_002985	Chemokine (C-C motif) ligand 5 (CCL5)	1.44	0.293668
NM_005125	Copper chaperone for superoxide dismutase (CCS)	−1.06	0.544413
NM_000397	Cytochrome b-245, beta polypeptide (CYBB)	2.30	0.054597
NM_134268	Cytoglobin (CYGB)	−1.27	0.401843
NM_175940	Dual oxidase 1 (DUOX1)	3.44	0.014514
NM_014080	Dual oxidase 2 (DUOX2)	2.76	0.162189
NM_004417	Dual specificity phosphatase 1 (DUSP1)	1.98	0.290027
NM_000502	Eosinophil peroxidase (EPX)	2.42	0.021458
NM_001979	Epoxide hydrolase 2, cytoplasmic (EPHX2)	1.43	0.070129
NM_002032	Ferritin, heavy polypeptide 1 (FTH1)	1.84	0.026055
NM_021953	Forkhead box M1 (FOXM1)	1.37	0.330551
NM_001450	Four and a half LIM domains 2 (FHL2)	1.76	0.009407
NM_000169	Galactosidase, alpha (GLA)	1.61	0.076094
NM_001498	Glutamate-cysteine ligase, catalytic subunit (GCLC)	3.60	0.032331
NM_002061	Glutamate-cysteine ligase, modifier subunit (GCLM)	4.86	0.004368
NM_000581	Glutathione peroxidase 1 (GPX1)	1.81	0.011334
NM_002083	Glutathione peroxidase 2 (GPX2)	4.18	0.042426
NM_002084	Glutathione peroxidase 3 (GPX3)	−2.15	0.042700
NM_002085	Glutathione peroxidase 4 (GPX4)	1.40	0.095091
NM_001509	Glutathione peroxidase 5 (GPX5)	2.17	0.193101
NM_000637	Glutathione reductase (GSR)	1.50	0.336807
NM_000852	Glutathione S-transferase pi 1 (GSTP1)	2.58	0.001655
NM_000178	Glutathione synthetase (GSS)	1.27	0.045122
NM_001513	Glutathione transferase zeta 1 (GSTZ1)	−1.09	0.662221
NM_005345	Heat shock 70kDa protein 1A (HSPA1A)	−1.11	0.763232
NM_001017963	Heat shock protein 90kDa alpha (cytosolic), class A member 1 (HSP90AA1)	3.34	0.007239
NM_002133	Heme oxygenase (decycling) 1 (HMOX1)	3.37	0.016069
NM_006121	Keratin 1 (KRT1)	2.00	0.229629
NM_006151	Lactoperoxidase (LPO)	−15.51	0.021233
NM_000242	Mannose-binding lectin (protein C) 2, soluble (MBL2)	2.12	0.189694
NM_005954	Metallothionein 3 (MT3)	2.38	0.347306
NM_012331	Methionine sulfoxide reductase A (MSRA)	1.01	0.840076
NM_002437	MpV17 mitochondrial inner membrane protein (MPV17)	1.31	0.039592
NM_000250	Myeloperoxidase (MPO)	−7.09	0.063002
NM_005368	Myoglobin (MB)	1.70	0.126031
NM_000903	NAD(P)H dehydrogenase, quinone 1 (NQO1)	7.99	0.006945
NM_016931	NADPH oxidase 4 (NOX4)	1.10	0.664407
NM_024505	NADPH oxidase, EF-hand calcium binding domain 5 (NOX5)	1.36	0.430648
NM_000265	Neutrophil cytosolic factor 1 (NCF1)	1.77	0.782783
NM_000433	Neutrophil cytosolic factor 2 (NCF2)	2.61	0.039859
NM_000625	Nitric oxide synthase 2, inducible (NOS2)	17.62	0.024602
NM_181782	Nuclear receptor coactivator 7 (NCOA7)	1.32	0.198973
NM_002452	Nudix (nucleoside diphosphate linked moiety X)-type motif 1 (NUDT1)	−1.00	0.700534
NM_020992	PDZ and LIM domain 1 (PDLIM1)	1.89	0.000223
NM_002574	Peroxiredoxin 1 (PRDX1)	3.22	0.016044
NM_005809	Peroxiredoxin 2 (PRDX2)	−1.03	0.809541
NM_006793	Peroxiredoxin 3 (PRDX3)	2.38	0.029921
NM_006406	Peroxiredoxin 4 (PRDX4)	−1.27	0.715809
NM_181652	Peroxiredoxin 5 (PRDX5)	3.06	0.018082
NM_004905	Peroxiredoxin 6 (PRDX6)	1.47	0.056072
NM_022126	Phospholysine phosphohistidine inorganic pyrophosphate phosphatase (LHPP)	1.89	0.032497
NM_183079	Prion protein (PRNP)	1.42	0.130447
NM_012212	Prostaglandin reductase 1 (PTGR1)	1.90	0.018686
NM_000962	Prostaglandin-endoperoxide synthase 1 (PTGS1)	1.06	0.773287
NM_000963	Prostaglandin-endoperoxide synthase 2 (PTGS2)	1.01	0.915271
NM_014245	Ring finger protein 7 (RNF7)	1.51	0.041667
NM_005410	Selenoprotein P, plasma, 1 (SEPP1)	1.14	0.374880
NM_203472	Selenoprotein S (VIMP)	−1.08	0.688003
NM_003900	Sequestosome 1 (SQSTM1)	−1.12	0.506208
NM_003122	Serine peptidase inhibitor, Kazal type 1 (SPINK1)	−1.71	0.461753
NM_012237	Sirtuin 2 (SIRT2)	1.03	0.861359
NM_014331	Solute carrier family 7 (SLC7A11)	2.15	0.028303
NM_080725	Sulfiredoxin 1 (SRXN1)	−1.26	0.328914
NM_000454	Superoxide dismutase 1, soluble (SOD1)	2.62	0.010116
NM_000636	Superoxide dismutase 2, mitochondrial (SOD2)	1.49	0.049991
NM_003102	Superoxide dismutase 3, extracellular (SOD3)	−3.23	0.002625
NM_003019	Surfactant protein D (SFTPD)	−1.14	0.651687
NM_003329	Thioredoxin (TXN)	2.48	0.039142
NM_003330	Thioredoxin reductase 1 (TXNRD1)	2.44	0.017214
NM_006440	Thioredoxin reductase 2 (TXNRD2)	−1.05	0.969389
NM_000547	Thyroid peroxidase (TPO)	2.96	0.027426
NM_003319	Titin (TTN)	2.19	0.002894
NM_024108	Trafficking protein particle complex 6A (TRAPPC6A)	1.15	0.337397
NM_003355	Uncoupling protein 2 (UCP2)	3.47	0.001925

**Table 3:** Confirmation of the expression levels of some randomly selected genes by customized PCR microarray.

Category	No.	Gene Name	Fold Regulation	*p*-Value
nonDEGs	1	Peroxiredoxin 4 (PRDX4)	−1.289	0.02521
	2	Dual specificity phosphatase 1 (DUSP1)	−1.7163	0.00599
DEGs	1	Lactoperoxidase (LPO)	−5.1107	0.00001 ****
	2	Superoxide dismutase 3, extracellular (SOD3)	−3.1359	1 × 10^6^ ****
	3	Nitric oxide synthase 2, inducible (NOS2)	4.7613	0.01469 *
	4	Heme oxygenase (decycling) 1 (HMOX1)	2.4445	0.00237 **

* *p* < 0.05, ** *p* < 0.01, and **** *p* < 0.0001 versus control.

**Table 4:** (a) The putative OxS status based on analysis of the DEGs identified in CRSsNP; (b) The putative nitrosative stress (NsS) status based on analysis of the DEGs identified in CRSsNP.

(a)		
**Category**	**Putative OxS Status**	**DEGs Involved**
Increase in Antioxidant Defense and Redox Balance	↑ ROS detoxification (especially H_2_O_2_ and lipid hydroperoxides)	↑ PRDX1/3/5, GPX2, SOD1, TXN, and TXNRD1
	↑ Glutathione synthesis and cystine import → robust GSH system	↑ GCLC, GCLM, and SLC7A11
	↑ Detoxification of quinones and electrophilic compounds	↑ NQO1 and GSTP1
ROS Production/Inflammation	↓ Extracellular ROS clearance → ↑ extracellular oxidative stress risk	↓ GPX3 and SOD3
	↑ ROS generation (hydrogen peroxide, nitric oxide and superoxide)	↑ DUOX1, NOS2, and NCF2
	↑ Oxidative burst activity	↑ EPX
	↓ Classic peroxidase activity linked to antimicrobial defense	↓ LPO
Stress Response and Protein Quality Control	↑ Heme catabolism, cytoprotection, anti-inflammatory signaling and protein folding	↑ HMOX1, HSP90AA1, and BAG2
Energy Metabolism and Mitochondrial Function	↓ Mitochondrial membrane potential and ROS production at mitochondria	↑ UCP2
Other (Extracellular/Structural)	Associated with muscle structural integrity and plasma antioxidant capacity	↑ ALB and TTN
(b)		
**Process**	**Putative NsS Status**	**DEGs Involved**
NO/RNS Production	↑ NO, superoxide, peroxynitrite → nitration/S-nitrosylation	↑ NOS2, NCF2, and DUOX1
Protein (De)nitrosylation	Removal of nitrosyl groups from proteins	↑ TXN and TXNRD1
S-Nitrosothiol Formation	Buffer/trans-nitrosylation	↑ GCLC, GCLM, SLC7A11, GSTP1, and NQO1
ROS Regulation of RNS	Modulates ROS/RNS interplay	↑ PRDXs, GPX2, and SOD1
NOS2 Stabilization	Sustains NO production	↑ HSP90AA1
Extracellular Vulnerability	Increased extracellular nitrosative stress	↓ GPX3 and SOD3

↑: increase; ↓: decrease.

## Data Availability

The original contributions presented in this study are included in the article/Supplementary Material. Further inquiries can be directed to the corresponding author.

## Supplementary Materials

The following supporting information can be downloaded at https://www.mdpi.com/article/10.3390/antiox14111292/s1. Table S1: The patients’ information and tissue samples used in the analysis. Table S2: The unique/overlapping DEGs of CRSsNP and -wNP and their biological functions and possible clinical implications. Figure S1: The protein interaction network and gene ontology (GO) enrichment analysis of the overall 27 and 23 DEGs of CRSsNP and -wNP by the STRING database.
