# Supplementary material for: Differential Expression of Antioxidant and Oxidant Pathways in Chronic Rhinosinusitis Without Nasal Polyps
Source: Antioxidants (Basel). 2025 Oct 28;14(11):1292. doi: 10.3390/antiox14111292 (PMC12649171; doi:10.3390/antiox14111292)
Supplement: Supplementary file 1 [file antioxidants-14-01292-s001.zip › antioxidants-3905143-supplementary.pdf]

## Supplementary data

**Methods**

Formal power analysis

A formal power analysis is a statistical method to calculate the minimum sample size needed to detect a real effect in a study with a given confidence level. Briefly, for equal-sized groups, an approximate sample size per group is calculated by the following equation:

$$n = \frac{(Z_{1-\alpha/2}+Z1-\beta)^2 \cdot 2\sigma^2}{\Delta^2}$$

Where:

$Z_{1-\alpha/2}$ is the standard normal quantile for two-sided $\alpha$.

$Z_{1-\beta}$ is the standard normal quantile corresponding to the desired power.

$\sigma$ = standard deviation of log2-expression.

$\Delta$ = difference in means on log2 scale.

If one predefined gene is targeted, we can use a standard two-sample test with $\alpha$=0.05. However, this study was trying to detect *any* DEGs among the 84 genes (multiple testing) and therefore family-wise error by Bonferroni is used, so $\alpha$_adj = 0.05 / 84 ≈ 0.000595238. A two-sided test was performed; therefore, we used $\alpha$_adj/2 for the Z quantile. The desired power was set to 0.80 (β = 0.20). The target effect was 2-fold change, thus Δ = log2(2) = 1.0. The σ (log2 expression) was 0.5. Accordingly, the n was calculated to be 9.14, which was rounded up to 10 subjects per group. However, if σ (log2 expression) was 0.75 and 1, the effect sizes (n) were calculated to be 21 and 37 by rounding up, respectively.

Table S1 **The patients' information and tissue samples used in the analysis**

| Analysis | Gene targeted | Group | Ages (min-max) | Sex (male/female) | Sample provenance |
|---|---|---|---|---|---|
| PCR microarray | 84 oxidative stress genes (n=6) | CTL | 23 – 74 | 3/3 | 6 control and 6 CRSsNP patients (independent patients*) |
| | | | 38.7 ± 18.60 | | |
| | | SNP | 36-62 | 2/4 | |
| | | | 44.0 ± 10.80 | | |
| Customized PCR array | PRDX4, DUSP1, LPO, SOD3, NOS2, HMOX1 (n=18) | CTL | 17-60 | 14/4 | 18 control and 18 CRSsNP patients (new and independent patients*) |
| | | | 37.11 ± 12.41 | | |
| | | SNP | 19-68 | 10/8 | |
| | | | 48.06 ± 12.57 | | |
| RT-PCR# | HSP90AA1 and GPX3 (n=5) | SNP | 20-52 | 2/3 | The total RNAs were prepared from the same tissues used for PCR microarray and the customized PCR microarray and were "randomly" used in the RT-PCR analysis. |
| | | | 33.6±13.2 | | |
| | | SNP | 36-64 | 2/3 | |
| | | | 51±12.1 | | |
| | NOS2 and SOD3 (n=18) | CTL | 17-60 | 14/4 | |
| | | | 37.1±12.1 | | |
| | | SNP | 19-68 | 10/8 | |
| | | | 48.1±12.2 | | |
| | HMOX-1 (n=16) | CTL | 22-60 | 13/3 | |
| | | | 37.4±11.1 | | |
| | | SNP | 19-68 | 10/6 | |
| | | | 47.1±12.6 | | |
| WB analysis# | NOS2 (n=8-10) | CTL | 18-72 | 6/2 | The total cell lysates for WB were prepared from the same tissues used for the customized PCR array and were "randomly assigned" to the WB analysis. |
| | | | 35.4±17.1 | | |
| | | SNP | 29-84 | 4/6 | |
| | | | 52.7±16.7 | | |
| | HMOX-1, SOD3 and LPO (n=10) | CTL | 23-72 | 8/2 | |
| | | | 46.1±17.3 | | |
| | | SNP | 29-84 | 8/2 | |
| | | | 59.6±15.4 | | |
| IHC | 4-HNE and 3-nitrotyrosine expression (n=9) | CTL | 23-72 | 7/2 | The tissue samples were prepared from the same tissues used for the customized PCR array and were "randomly" used for IHC analysis. |
| | | | 43±15.4 | | |
| | | SNP | 29-84 | 7/2 | |
| | | | 58.4±15.8 | | |

[*] 24 independent patients in total were used in this study.

[#] Same tissue samples of patients from the PCR and customized PCR microarray were randomly used in these analyses.

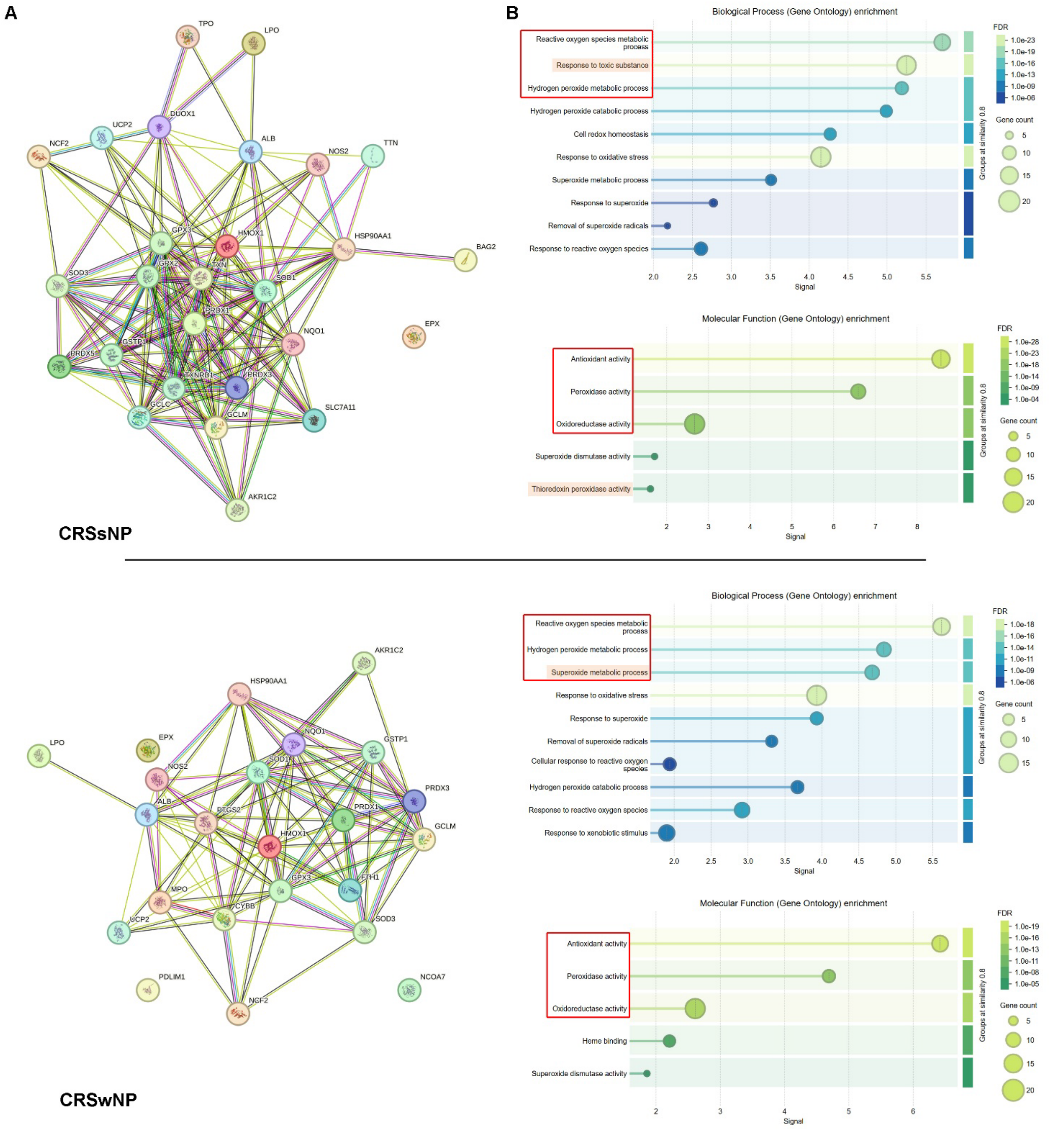


**Figure S1 The protein interaction network and gene ontology (GO) enrichment analysis of the overall 27 and 23 DEGs of CRSsNP and -wNP by the STRING database.** The overall 27 DEGs of CRSsNP (current study) and 23 DEGs of CRSwNP [1] were subjected to analysis by the STRING database (Version 12.0) [2]. The explanation of the nodes, lines, and edges in the interaction network has been provided in Figure 7. The top 3 in the GO enrichment analysis are highlighted by a red box frame and the differences between CRSsNP and -wNP are indicated by an orange-color background text.

**Table S2** The unique/overlapping DEGs of CRSsNP and -wNP and their biological functions and possible clinical implications

| | **CRSsNP** | **CRSwNP** | **CRSsNP vs -wNP** |
|---|---|---|---|
| **Unique/Overlapping DEGs found in PCR microarray** | TXN, TXNRD1, GPX2, GCLC, PRDX5, SLC7A11, DUOX1, TPO, BAG2, and TTN ↑ | 1. COX-2, CYBB, FTH1, NCOA7, and PDLIM1 ↑<br>**2.** MPO ↓ | 1. ALB, AKR1C2, EPX, GCLM, GSTP1, HSP90AA1, HMOX1, NQO1, NCF2, NOS2, PRDX1, PRDX3, SOD1, and UCP2 ↑<br>2. GPX3, LPO, and SOD3 ↓ |
| **Biological functions** | 1. Glutathione & thioredoxin axis: TXN, TXNRD1, GCLC, GPX2, PRDX5, SLC7A11 → Intracellular antioxidant buffering and maintaining redox homeostasis<br>2. Specialized processes:<br>• DUOX1, TPO: thyroid hormone biosynthesis, ROS generation<br>• BAG2: (co-)chaperone in protein quality control<br>• TTN: structural muscle protein (likely less central) | 1. Inflammation and ROS production:<br>• COX-2 (PTGS2): pro-inflammatory prostaglandins (PGs)<br>• MPO: neutrophil enzyme, generates hypochlorous acid (potent oxidant)<br>• CYBB: NOX2, superoxide production in phagocytes<br>2. Iron metabolism & stress (iron storage, ferroptosis regulation) and immune regulation: FTH1<br>3. Plays a role in redox homeostasis and may reduce ROS; also modulates interferon, hinting at roles in innate immunity: NCOA7<br>4. Cytoskeletal/ structural: (may influence inflammation through cytoskeleton and NF-κB signaling in immune cells): PDLIM1 | 1. Antioxidants & ROS detoxifiers: PRDX1, PRDX3, SOD1, SOD3, GPX3, HMOX1, NQO1, GCLM, GSTP1<br>2. Iron/heme metabolism: HMOX1, LPO, EPX<br>3. Classic peroxidase activity linked to antimicrobial defense: LPO<br>4. Nitric oxide/ROS signaling: NOS2, NCF2<br>5. Stress response chaperones: HSP90AA1<br>6. Metabolic/transport: ALB (serum transport), AKR1C2 (steroid/aldehyde metabolism)<br>7. Mitochondrial regulation: UCP2 (controls ROS production) |
| **Possible OxS effects** | Intracellular antioxidant defense, thioredoxin/ glutathione system, thyroid processes | Immune-driven inflammation, ROS production, and iron metabolism | General oxidative stress, NO signaling, and redox imbalance |
| **Clinical implications** | 1. Disease subtype may show redox imbalance, metabolic stress sensitivity, or thyroid dysfunction<br>2. Might benefit from therapies aimed at enhancing glutathione/thioredoxin pathways (e.g., N-acetylcysteine, thiol donors) | 1. Disease subtype may show chronic inflammation, PG-mediated pain/fever, and iron dysregulation<br>2. Might benefit more from anti-immune and inflammatory drugs (COX-2 inhibitors and NOX2 inhibitors) | Core oxidative/inflammatory stress is present in both diseases |

## References

1. Tsai, Y.J.; Hsu, Y.T.; Ma, M.C.; Wu, C.K.; Luo, S.D.; Wu, W.B. Transcriptomic analysis of genes associated with oxidative stress in chronic rhinosinusitis patients with nasal polyps: Identifying novel genes involved in nasal polyposis. *Antioxidants (Basel, Switzerland)* **2022**, *11*.
2. Szklarczyk, D.; Kirsch, R.; Koutrouli, M.; Nastou, K.; Mehryary, F.; Hachilif, R.; Gable, A.L.; Fang, T.; Doncheva, N.T.; Pyysalo, S., *et al.* The string database in 2023: Protein-protein association networks and functional enrichment analyses for any sequenced genome of interest. *Nucleic acids research* **2023**, *51*, D638-d646.
